# Correlation of Ventilation-Perfusion (V/Q) Scan Results as Compared with Clinical Probability of Pulmonary Embolism in African American Population

**DOI:** 10.7759/cureus.1353

**Published:** 2017-06-14

**Authors:** Fasil Tiruneh, Ahmad Awan, Nicole Hunt, Nahom Tegegn, Daniel Larbi

**Affiliations:** 1 Department of Internal Medicine, Howard University Hospital

**Keywords:** pulmonary embolism, african-american, wells clinical probability, ventilation-perfusion (v/q) scan

## Abstract

Introduction

Current guidelines suggest the use of the more specific Wells' score could safely reduce the number of unnecessary scans. There is a lack of research to support whether these guidelines apply to the African American population. This study aims to evaluate the correlation of clinical pretest probability of pulmonary embolism (PE) with ventilation-perfusion (V/Q) scan results in a predominantly African American population and to test whether current guidelines based on studies conducted in other populations hold true in this group.

Material and Methods

A retrospective descriptive study to determine the diagnostic utility of the V/Q scan was conducted among patients who were seen during January 2012 to January 2016. The study population included patients who underwent a V/Q scan for evaluation of PE. One hundred and seventy-five charts were reviewed and 49 were excluded due to poor quality data. A review of the initial history, as well as discharge summaries, was performed. Wells' probability of PE was compared with the results of the scan. Laboratory tests and imaging studies were reviewed and analyzed.

Result

The median age of the study population was 63.02 ± 16.12 years. The majority of the study population, 121 patients (92.4%), was African American. Sixty-four (48.9%) VQ scans were done for a low clinical probability for pulmonary embolism as defined by the Wells' clinical score. The most common clinical presentations were shortness of breath (SOB) - 74 (58%), leg pain or swelling - 39 (29.8%), chest pain - 36 (27.4%), and syncope - 4 (3.1%). Sixty-two (96.9 %) patients with low clinical probability had low probability VQ scans (P = 0.03). Among the patients who underwent CT angiography and V/Q scanning, a low probability scan was noted in 25 patients with no pulmonary embolism on CT (96.2 %) (P = 0.006).

Conclusions

This study showed a strong correlation between low clinical probability and low probability V/Q scans and its utility to safely rule out PE in a predominantly black population. Studies conducted in other populations have detected similar findings.

## Introduction

Ventilation-perfusion (V/Q) scans are ordered primarily to assess the probability of acute pulmonary embolus. This study is preferable over computerized tomography angiography (CTA) for patients with contrast allergy, renal failure, or those too large for the CT gantry or bed. This study may also be preferable in young patients (especially women) due to lesser radiation dose and those with clear lungs on x-ray [1].

The clinical probability of pulmonary emboli (PE) should be considered when factoring in V/Q scan interpretations as pre-imaging probability affects the accuracy of the interpretation. The most common clinical criteria for determining the pre-imaging probability of PE is the Wells' score. Many clinicians consider a negative d-dimer result, combined with a low-pretest probability of PE by the Wells' criteria, to be negative for acute PE, thus preventing the need for further assessment using imaging [2-3].

A high-probability scan usually indicates a pulmonary embolism, but only a minority of patients with pulmonary embolism have a high-probability scan. A history of pulmonary embolism decreases the accuracy of diagnoses based on high probability scans. A low-probability scan with a strong clinical impression that pulmonary embolism is not likely makes the possibility of pulmonary embolism remote. Near-normal/normal lung scans make the diagnosis of acute pulmonary embolism very unlikely. An intermediate-probability (indeterminate) scan is not of help in establishing a diagnosis. In the Prospective Investigation of Pulmonary Embolism Diagnosis (PIOPED) study, the scan, combined with clinical assessment, permitted a noninvasive diagnosis or exclusion of acute pulmonary embolism for a minority of patients [4].

## Materials and methods

A retrospective descriptive study to determine the diagnostic utility of V/Q scan was conducted among patients who were seen between January 2012 to January 2016. Ethical clearance to conduct the research was granted by the Office of Regulatory Research Compliance of Howard University (approval #FW A00000891).

The study population included patients who underwent a V/Q scan for evaluation of pulmonary embolism. One hundred and seventy-five charts were reviewed and 49 were excluded due to poor quality data. A review of the initial history and physical progress notes, as well as discharge summaries, was performed. Clinical presentation and Wells' probability of pulmonary embolism were compared with the results of the scan. Laboratory tests, including d-dimer, as well as imaging studies, including CTA and Doppler studies, were reviewed and analyzed.

## Results

We collected data on 131 patients who underwent V/Q scan for evaluation of pulmonary embolism. The median age of the study population was 63.02 ± 16.12 years. However, the age ranges between 15 and 96 years. The majority of the study population (121 patients) (92.4%) was African American with a male to female ratio of 0.8. Ethnicity was not seen to influence the result of the study [Table 1].

**Table 1:** Demographic Distribution of Patients Ethnicity and Gender by Result of V/Q Scan The table shows 108 African Americans (93.1%) have a low probability V/Q scan. High probability V/Q scan was noted in five African American patients (4.3%) (P = 0.71). V/Q: ventilation-perfusion scans; a: P-value 0.71; b: P-value 0.99; n: number

		Low probability V/Q scan n (%)	Intermediate probability V/Q scan n (%)	High probability V/Q scan n (%)	Total n (%)
GENDER	Male	51 (92.7%)	2 (3.6%)	2 (3.6%)	55 (100.0%)^a^
	Female	67 (94.4%)	1 (1.4%)	3 (4.2%)	71 (100.0%)
ETHNICITY	African American	108 (93.1%)	3 (2.6%)	5 (4.3%)	116 (100.0%)^b^
	Hispanic	6 (100.0%)	0 (0.0%)	0 (0.0%)	6 (100.0%)
	White	3 (100.0%)	0 (0.0%)	0 (0.0%)	3 (100.0%)
	Others	1 (100.0%)	0 (0.0%)	0 (0.0%)	1 (100.0%)

Sixty-four (48.9%) V/Q scans were done for a low clinical probability for pulmonary embolism as defined by the Wells' clinical score. High probability Wells' score was noted among 15 of the patients (11.5%) for whom a V/Q scan was performed. The high probability V/Q scan was observed in more females than males (4.2 and 3.6%, respectively). However, the difference was not statistically significant (P = 0.71).

**Figure 1:**
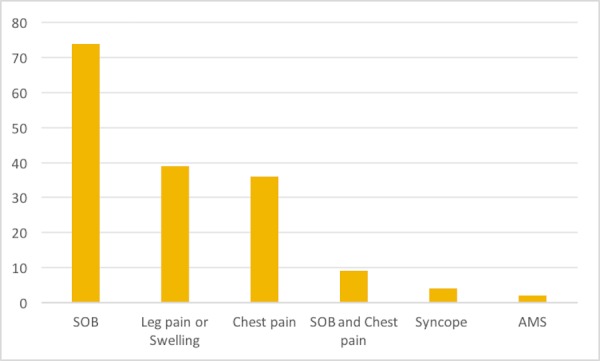
Bar chart showing frequency of clinical presentation of study population SOB: shortness of breath

The most common clinical presentations were shortness of breath (SOB) in 74 patients (58%), leg pain or swelling in 39 patients (29.8%), chest pain in 36 patients (27.4%), and syncope in four patients (3.1%). Recent immobilization in 14 patients (11.9%), previous deep venous thrombosis (DVT) in 22 patients (12.2%), and a history of malignancy in 15 patients (2.2%) were observed. One patient (0.8%) had hemoptysis, but this was noted to be rare among patients who underwent a V/Q study to rule out PE. Leg swelling was noted on physical examination in 39 patients (29.8%). PE was the number one diagnosis in 27 patients (20%) prior to the scan (Table 2) (Figure 1).

**Table 2:** Clinical Presentation of Patients by Results of V/Q scans The most common clinical presentation was SOB, leg pain/swelling, chest pain, and syncope. No statistically significant differences were noted between individual presenting symptoms and V/Q scan results. V/Q: ventilation-perfusion scans; SOB: shortness of breath P-values: a: 0.81, b: 0.90; c: 0.96

		Low probability V/Q scan n (%)	Intermediate probability V/Q scan n (%)	High probability V/Q scan n (%)	Total n (%)
CLINICAL PRESENTATION	SOB	66 (89.2%)	3 (4.1%)	5 (6.8%)	74 (100.0%)^a^
	Leg pain or swelling	36 (92.3%)	1 (2.6%)	2 (5.1%)	39 (100.0%)^b^
	Chest pain	36 (100.0%)	0 (0.0%)	0 (0.0%)	36 (100.0%)
	SOB and Chest pain	9 (100.0%)	0 (0.0%)	0 (0.0%)	9 (100.0%)
	Syncope	4 (100.0%)	0 (0.0%)	0 (0.0%)	4 (100.0%)
	Altered mental status	2 (100.0%)	0 (0.0%)	0 (0.0%)	2 (100.0%)
	Hemoptysis	1 (100.0%)	0 (0.0%)	0 (0.0%)	1 (100.0%)^c^

Among patients with a high clinical probability of pulmonary embolism by the Wells' criteria, shortness of breath was the main presenting symptom. More than half (58.8 %) of high probability scans were done for patients with shortness of breath. A high probability scan result was not seen among patients who presented with chest pain alone. However, the observation was not statistically significant (P = 0.88). Tachycardia was noted in four of five patients (80%) of high probability V/Q scans as compared to one out of 20 patients (5%) with normal heart rate (P = 0.003).

The study showed no statistically significant difference between recent immobilization and V/Q scan results (P = 0.67). A previous history of malignancy was associated with a higher number of intermediate and high probability V/Q scans as compared with patients with no history of malignancy (18.8% vs 4.5%, respectively), and the difference was noted to be statistically significant (P = 0.03). A low probability scan was noted in 105 patients (95.5%) with no previous history of malignancy versus 13 patients (81.3%) with a previous malignancy. A higher occurrence of high probability V/Q scan was observed in patients with previous history of DVT (9.1%) as compared with no previous DVT (2.9%). However, the association was not significant (P = 0.64). Among patients with pulmonary embolism as the primary diagnosis, 88% had a low probability scan, while a 95% low probability scan was seen in patients with other diagnoses (P = 0.12) (Table 3).

**Table 3:** Clinical Presentation of Patients by Results of V/Q Scans No statistically significant difference was found between recent immobilization and VQ scan result (p = 0.689). A previous history of malignancy was associated with a higher number of intermediate and high (p = 0.031). DVT: deep vein thrombosis; PE: pulmonary embolus; V/Q: ventilation-perfusion scans. p-Values: a: 0.003; b: 0.689; c: 0.031; d: 0.648; e: 0.12

		Low probability V/Q scan ​ n (%)		Intermediate probability V/Q scan n (%)	High probability V/Q scan n (%)	Total n (%)	
Heart Rate	Normal heart rate	0 (0.0%)	1 (1.2%)		85 (100.0%)	86 (100%)	
	Tachycardia	1 (3.4%)	4 (13.8%)		29 (100.0%)	34 (100%) ^a^	
Recent Immobilization	No immobilization	93 (93.0%)	3 (3.0%)		4 (4.0%)	100 (100.0%)^b^	
	Recent immobilization	12 (92.3%)	0 (0.0%)		1 (7.7%)	13 (100.0%)	
H/O Malignancy	No malignancy	105 (95.5%)	2 (1.8%)		3 (2.7%)	110 (100.0%)^c^	
	History of malignancy	13 (81.3%)	1 (6.3%)		2 (12.5%)	16 (100.0%)	
H/O Previous DVT	No previous DVT	97 (94.2%)	3 (2.9%)		3 (2.9%)	103 (100.0%)^d^	
	Previous DVT	20 (90.9%)	0 (0.0%)		2 (9.1%)	22 (100.0%)	
Diagnosis	PE not as number one diagnosis	96 (95.0%)	1 (1.0%)		4 (4.0%)	101 (100.0%)^e^	
	PE as number one diagnosis	22 (88%)	2 (8%)		1 (4%)	25 (100%)	

**Figure 2:**
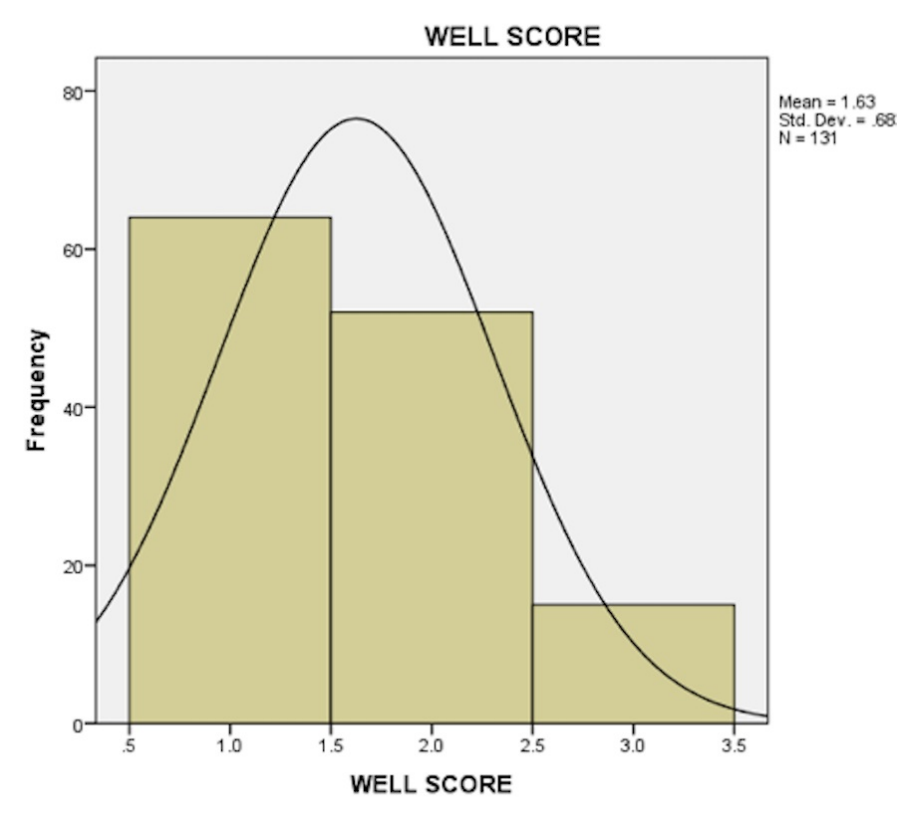
Bar chart showing the frequency of Wells' score results among the study population

**Figure 3:**
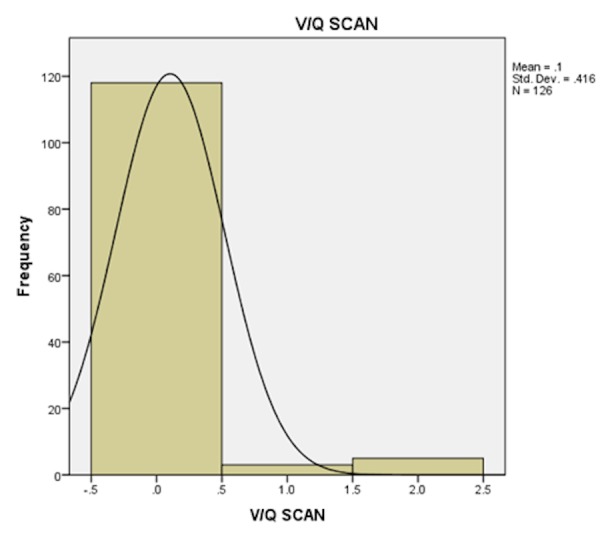
Bar chart showing the frequency of V/Q scan results among the study population V/Q: ventilation-perfusion

Only 10 patients (8.5%) with low probability VQ scan had a high clinical probability for pulmonary embolism. Sixty-two patients (96.9%) with low clinical probability had a low probability V/Q scan. Three (23.1 %) patients with high clinical probability had an intermediate or high probability V/Q scan. Patients with moderate Wells' scores were noted to have low probability scan in 93.9% and a high probability scan in 6.1%. Differences seen with clinical Wells' scores were statistically significant (P =0.03) (Table 4) (Figures 2-3).

**Table 4:** Correlation Between the Clinical Pretest Probability with V/Q Scan Probability Sixty-two patients (96.9%) with low clinical probability had a low probability V/Q scan (P = 0.03). V/Q: ventilation-perfusion scans; P-value a: 0.03

		Low Probability V/Q scan n (%)	Intermediate Probability V/Q scan n (%)	High Probability V/Q scan n (%)	Total n(%)
Wells' score	Low clinical probability	62 (96.9%)	2 (3.1%)	0 (0.0%)	64 (100.0%)^a^
	Moderate clinical probability	46 (93.9%)	0 (0.0%)	3 (6.1%)	49 (100.0%)
	High clinical probability	10 (76.9%)	1 (7.7%)	2 (15.4%)	13 (100.0%)

No clear association was noted between a troponin level and V/Q scan probability. The majority of patients with intermediate and high probability scans have no leukocytosis (94 (82.1%) vs 21 (17.9%) (P = 0.71)). Elevated creatinine levels of greater than or equal to 1.5 mg/dl were seen in 74/117 (63.5%) of the study population and were the main indication to prefer a V/Q scan to rule out PE as opposed to CTA. D-dimer test was done in 43/131 patients. Among patients with normal d-dimer tests, the result of the V/Q scan was a low probability in 100%. Elevated d-dimer tests were reported in 38/43 (89.4%) of patients in which 7.3% showed intermediate or high probability V/Q scan (P = 0.82).

The presence of deep venous thrombosis (DVT) on Doppler ultrasound was another parameter that was assessed with the results of VQ scans. In 62 patients (47.7%), a Doppler scan to rule out DVT was done prior to the scan and was positive in 11 (8.5%) of the patients. Of the patients with DVT, 9.1% had a high probability V/Q scan versus 4% in patients with no DVT (P = 0.76) (Table 5).

**Table 5:** Correlation Between D-dimer, DVT, and CTA Findings as Compared with V/Q Scan Probability High probability scans were noted in 33.3% of patients who had a positive computed tomography angiography for pulmonary embolism (P = 0.006). COPD: chronic obstructive pulmonary disease; CT: computed tomography; DVT: deep vein thrombosis; ILD: interstitial lung disease; PE: pulmonary embolus; US: ultrasound; V/Q: ventilation-perfusion scans P-values:  a: 0.82; b: 0.76; c: .0.006

		Low probability V/Q scan n(%)	Intermediate probability V/Q scan n (%)	High probability V/Q scan n (%)	Total n (%)
D-Dimer	Not elevated	5 (100.0%)	0 (0.0%)	0 (0.0%)	5 (100.0%)^a^
	Elevated	38 (92.7%)	2 (4.9%)	1 (2.4%)	41 (100.0%)
Doppler US	No DVT	46 (92.0%)	2 (4.0%)	2 (4.0%)	50 (100.0%)^b^
	DVT	10 (90.9%)	0 (0.0%)	1 (9.1%)	11 (100.0%)
	No PE	25 (96.2%)	1 (3.8%)	0 (0.0%)	26 (100.0%)^c^
CT Finding	Pneumonia	4 (100.0%)	0 (0.0%)	0 (0.0%)	4 (100%)
	Effusion	4 (100.0%)	0 (0.0%)	0 (0.0%)	4 (100%)
	COPD	1 (50.0%)	0 (0.0%)	1 (50.0%)	2 (100%)
	ILD	1 (100.0%)	0 (0.0%)	0 (0.0%)	1 (100.0%)
	PE	2 (66.7%)	0 (0.0%)	1 (33.3%)	3 (100.0%)
	Limited study	2 (66.7%)	1 (33.3%)	0 (0.0%)	3 (100.0%)

CT of the chest was done in 44 patients (42.7%) following a negative VQ scan, and pneumonia and pleural effusion were the commonest diagnoses made. On the other hand, limited studies were accountable for ordering a VQ scan in three of the cases (2.5%). Pulmonary embolism was noted in 2.5% of patients who had a V/Q scan. Among patients who underwent CTA and V/Q scan, a low probability scan was noted in patients with no pulmonary embolism on CTA in 25 (96.2%). A high probability scan was noted in 33.3% of patients who had positive CT angiography for pulmonary embolism. The difference was statistically significant (P = 0.006) (Table 5).

## Discussion

The advantages of the V/Q scan are a lower radiation dose than computed tomography pulmonary angiogram (CTPA) and the lack of need for iodinated contrast; therefore, V/Q scanning is often considered as the preferred alternative chest imaging to CTPA. The effective radiation dose from a V/Q scan is 1.4 mSv compared to 10-12 mSv from routine CTPA [5-6]. The clinical probability assessment using the Wells' score provides greater accuracy in estimating the clinical probability score, independent of clinician's experience, and allows the option for alternative diagnosis. A study by Wells, et al. showed a pretest probability of low in 734 patients (3.4% with pulmonary embolism), moderate in 403 (27.8% with pulmonary embolism), and high in 102 (78.4% with pulmonary embolism) [7].

In the PIOPED II study, new dyspnea at rest or on exertion was the most frequent symptom in patients with pulmonary embolism and no prior cardiopulmonary disease (73%). Similarly, our study also showed dyspnea to be the commonest clinical presentation. Immobilization (bedrest within past month for the most of the day for ≥ three consecutive days) was the most frequent risk factor assessed in patients with pulmonary embolism. In our study, recent immobilization was noted in 12/105 patients. Among all patients with pulmonary embolism, calf swelling, plus pain with palpation of the deep veins, occurred in 32%. Our study also showed a relatively higher occurrence of high probability scan among patients with leg pain or swelling (5.1% vs 2.1%) [8]. Similar to our study population where hemoptysis was the least presenting symptom, in both the PIOPED and PIOPED II studies, hemoptysis, when present, occurred only in small amounts [9].

A study by Barghouth, et al. suggested low V/Q scan probability associated with low clinical probability could exclude PE in 43/45 cases (96%). In our study, the pretest probability was low in 96.6% of the low probability scans. This was consistent with most other studies demonstrating a strong correlation between low clinical probability and low probability V/Q scans and its utility to safely rule out PE. However, there was a relatively higher number of low probability scans (93.7%) than the Barghouth, et al., study (57%) [10]. Although the sensitivity of the d-dimer test is high, the specificity is not sufficiently high enough for the test to be diagnostic; however, d-dimer is a valuable tool in the exclusion of PE, as the negative predictive value of d-dimer is high [11].

A possible limitation of our study is the low prevalence of pulmonary embolism in our sample; this may suggest that most of the patients were at lower risk. Data recording was also not optimal in some patients. It is possible that the results of our study may not apply to a patient population with a higher prevalence of thromboembolic disease. However, it is expected to hold true in a population with a low probability of pulmonary embolism.

## Conclusions

Using the accepted guidelines in which a high pretest probability leads to further imaging and a low probability leads to a d-dimer blood test, use of the more specific Wells' score could safely reduce the number of unnecessary scans. The PIOPED II investigators recommend stratification of all patients with suspected pulmonary embolism according to an objective clinical probability assessment. Our study has reflected the importance of a negative d-dimer test combined with a low or moderate clinical probability to rule out PE. However, no statistically significant correlation was observed between elevated d-dimer and results of a V/Q scan. In view of this finding, we suggest other possible diagnoses should be explored, even in patients with elevated d-dimer tests.
